# Clinical and neuroimaging features as diagnostic guides in neonatal neurology diseases with cerebellar involvement

**DOI:** 10.1186/s40673-016-0039-1

**Published:** 2016-01-13

**Authors:** Jessica L. Klein, Monica E. Lemmon, Frances J. Northington, Eugen Boltshauser, Thierry A. G. M. Huisman, Andrea Poretti

**Affiliations:** Department of Neurology, The Johns Hopkins University School of Medicine, Baltimore, MD USA; Neurosciences Intensive Care Nursery Program, The Johns Hopkins University School of Medicine, Baltimore, MD USA; Department of Pediatrics, Medical University of South Carolina, Charleston, SC USA; Division of Pediatric Neurology, Department of Pediatrics, Duke University School of Medicine, Durham, NC USA; Division of Neonatology, Department of Pediatrics, The Johns Hopkins University School of Medicine, Baltimore, MD USA; Division of Pediatric Neurology, University Children’s Hospital, Zurich, Switzerland; Section of Pediatric Neuroradiology, Division of Pediatric Radiology, The Russell H. Morgan Department of Radiology and Radiological Science, The Johns Hopkins University School of Medicine, Charlotte R. Bloomberg Children’s Center, Sheikh Zayed Tower, Room 4174, 1800 Orleans Street, Baltimore, MD USA

**Keywords:** Cerebellum, Neonate, Neonatal neurology, Neuroimaging

## Abstract

Cerebellar abnormalities are encountered in a high number of neurological diseases that present in the neonatal period. These disorders can be categorized broadly as inherited (e.g. malformations, inborn errors of metabolism) or acquired (e.g. hemorrhages, infections, stroke). In some disorders such as Dandy-Walker malformation or Joubert syndrome, the main abnormalities are located within the cerebellum and brainstem. In other disorders such as Krabbe disease or sulfite oxidase deficiency, the main abnormalities are found within the supratentorial brain, but the cerebellar involvement may be helpful for diagnostic purposes. In In this article, we review neurological disorders with onset in the neonatal period and cerebellar involvement with a focus on how characterization of cerebellar involvement can facilitate accurate diagnosis and improved accuracy of neuro-functional prognosis.

## Background

In the last decade, remarkable progress has been made in neonatal neurology. The translation of this new information from the bench to the clinic has allowed for significant improvements in the diagnosis and treatment of neurological disorders of preterm and term neonates. At present, as many as 25 % of newborns in tertiary-level intensive care nurseries have primary neurological conditions and many more are at risk of neurologic injury [1].

The cerebellum accounts for only about 10 % of the brain volume, but contains approximately 50 billion neurons (more than 50 % of all the neurons of the brain) and 200 million input fibers. The cerebellum is highly connected with the cerebral hemispheres through numerous complex anatomical and functional cerebro-cerebellar circuits [2, 3]. The cerebro-cerebellar connections include the motor cortex and the supplementary motor area, the posterior and inferior parietal cortex, the superior temporal cortex, the prefrontal cortex, as well as the cingulate gyrus and the parahippocampal gyrus. The circuits of the cerebro-cerebellar connections are the likely structural basis for the important role of the cerebellum in motor and neurocognitive functions. In the last decades, understanding of cerebellar dysfunction has evolved from pure motor syndromes (impairments of gait, extremity coordination, speech, and ocular motor control) to more complex neurocognitive and neurobehavioral abnormalities that are collectively known as the “cerebellar cognitive affective syndrome” [4].

This review article focuses on disorders with cerebellar involvement that present during the neonatal period. Brainstem involvement has also been included because brainstem abnormalities often accompany cerebellar anomalies and cerebellar and brainstem embryological development is closely related. The diseases listed in this article have been selected from our clinical experience, consultation of textbooks on cerebellar disorders, neonatal neurology, neonatology, and neuroimaging, and a search of Pubmed with appropriate key words. The disorders have been broadly classified as inherited or acquired (Table 1). Selected cerebellar and brainstem malformations such as rhombencephalosynapsis, the majority of cases of Dandy-Walker malformation, and pontine tegmental cap dysplasia have been classified as hereditary disorders although the genetic background is still unknown. Their origin is most likely hereditary (genetic), not acquired based on the neuroimaging findings and suggested patho-mechanisms.

**Table 1:** Neonatal neurology diseases with clinical or neuroimaging cerebellar involvement

Group of diseases		Diseases	Diagnostic test
Inherited	Malformation	Dandy-Walker malformation	Brain MRI findings
		Joubert syndrome	Brain MRI findings
		Rhombencephalosynapsis	Brain MRI findings
		Pontocerebellar hypoplasias	Brain MRI findings, genetic analysis
		Congenital muscular dystrophy due to α-dystroglycanopathy	Muscle biopsy, genetic analysis
		Brainstem disconnection	Brain MRI findings
		Pontine tegmental cap dysplasia	Brain MRI findings
		Chiari type II malformation	Brain MRI findings
	Neurometabolic	Congenital disorders of glycosylation	Transferrin electrophoresis, genetic analysis
		Smith-Lemli-Opitz syndrome	Elevated 7-dehydrocholesterol, genetic analysis
		Non-ketotic hyperglycinemia	Elevated CSF glycine concentration and CSF-to-plasma glycine ratio, genetic analysis
		Maple syrup urine disease	Elevated branched-chain amino acids and branched-chain keto-acids in blood and urine
		Pyruvate dehydrogenase deficiency	Abnormal enzyme function, genetic analysis
		Sulfite oxidase deficiency	Elevated sulfite, thiosulfate, taurine, and S-sulfocysteine concentrations in urine, genetic analysis
	Neurodegenerative	Krabbe disease	Low galactocerebrosidase activity in peripheral leukocytes, genetic analysis
		Cockayne syndrome	Genetic analysis
		Pelizaeus-Merzbacher disease	Genetic analysis
		Aicardi-Goutières syndrome	Genetic analysis
		Congenital neuronal ceroid lipofuscinosis	Genetic analysis
	Spinocerebellar ataxias	SCA type 2	Genetic analysis
		SCA type 7	Genetic analysis
Acquired	Vascular	Cerebellar hemorrhage	Brain MRI findings
		Sinovenous thrombosis	Brain MRI findings
		cerebellar ischemic stroke	Brain MRI findings
	Disrupted development of the cerebellum in preterms		History of prematurity, brain MRI findings
	Hypoxic-ischemic injury		Brain MRI findings
	Infection	Congenital cytomegalovirus	Viral culture, PCR, or serology within the first weeks of life, later PCR for CMV DNA in dried blood of neonatal screening
		Herpes simplex	CSF viral culture or PCR
	Toxic	Glucocorticoids	Brain MRI findings, history of glucocorticoids therapy
	Teratogens	Alcohol	History, brain MRI findings
		Retinoic acid	History, brain MRI findings
		Misoprostol	History, brain MRI findings
	Tumors	Teratoma	Histology
		Medulloblastoma	Histology

*CMV* cytomegalovirus, *CSF* cerebrospinal fluid, *DNA* deoxyribonucleic acid, *MRI* magnetic resonance imaging, *PCR* polymerase chain reaction

The discussion of each disorder will emphasize how understanding of cerebellar involvement may facilitate diagnosis, elucidate pathogenesis, and affect long-term neurocognitive and behavioral outcomes of the affected preterm and term neonates. In addition, we provide checklists for selected clinical (Table 2) and neuroimaging (Table 3) findings. These checklists should facilitate making the correct diagnosis or narrow the differential diagnoses of neonatal neurological diseases with cerebellar involvement. These lists can be used as “memo-lists” and are not meant to be complete. Note that for a given condition a range of findings may occur and not all mentioned clinical and imaging findings have to be present.

**Table 2:** Neonatal symptoms and clinical findings that may facilitate the diagnosis of neonatal disorders with cerebellar involvement

Symptom/clinical finding	Diseases
Alopecia	RES
Breathing abnormalities	BD, CII, cerebellar hemorrhage, CNCL, JS, NKH, PCH 1/4, PDH
Contractures	CMD, CS, PCH 4
Corneal insensitivity	PTCD, RES
Dysmorphic features	CDG, CS, JS, RES, SLO, SOD
Dysphagia	BD, CII, CMD, PCH 1/2/6, PTCD
Dystonia	MSUD, PCH 2
Eye involvement (cataract, coloboma, retinitis, …)	CMD, CMV, CNCL, CS, JS
Facial palsy	BD, PTCD
Head titubation	JS
Hyporeflexia	CDG, PCH 1
Irritability	Cerebellar hemorrhage, Krabbe, sinovenous thrombosis
Kidney involvement	JS, SLO
Macrocephaly	Cerebellar hemorrhage, CII, DWM, RES, sinovenous thrombosis, tumor
Microcephaly	CMV, CNCL, PCH (mainly type 6), PDH, SLO
Nystagmus	PMD
Ophthalmoplegia	MSUD
Polydactyly	JS
Ptosis	SLO
Seizures	BD, cerebellar hemorrhage and stroke, CMD, CMV, CNCL, HII, HSV, MSUD, NKH, preterm disruption, PCH 6, PDH, SOD
Skeletal abnormalities	BD, PTCD, SLO
Stridor	CII, PMD
Unstable body temperature	BD
Weakness	CMD, PCH 1

*BD* brainstem disconnection, *CDG* congenital disorders of glycosylation, * CII* Chiari type II malformation, *CMD* congenital muscular dystrophy, *CMV* cytomegalovirus infection, *CNCL* congenital neuronal ceroid lipofuscinosis, *CS* Cockayne syndrome, *DWM* Dandy-Walker malformation, *HII* hypoxic-ischemic injury, *HSV* herpes simpex infection, *JS* Joubert syndrome, *MSUD* maple syrup urine disease, *NKH*, nonketotic hyperglycinemia, *PCH* pontocerebellar hypoplasia, *PDH* pyruvate dehydrogenase deficiency, *PMD* Pelizaeus-Merzbacher disease, *PTCD* pontine tegmental cap dysplasia, *RES* rhombencephalosynapsis, *SLO* Smith-Lemli-Opitz syndrome, *SOD* sulfite oxidase deficiency

**Table 3:** Neuroimaging findings that may facilitate the diagnosis of neonatal disorders with cerebellar involvement

Neuroimaging finding		Diseases
Calcifications		AGS, CMV, CS
Callosal agenesis/dysgenesis		CII, CMD, NKH, SLO
Cerebellar atrophy (global)		AGS, CDG, CNCL, CS
Cerebellar cysts		CMD, PCH1/2/6
Cerebellar hemispheres	Atrophy	Preterm disruption, PCH
	Dysplasia	CMD
	Hypoplasia	DWM, PCH
Cerebral atrophy		AGS, CNCL, CS
Dentate nuclei T2-hyperintense signal		Krabbe
Global cerebellar hypoplasia		BD, CDG, CMV, NKH, SOD, SLO
Global cerebral edema		SOD, HII
Malformation of cortical development		CMD, CMV, JS
Molar tooth sign		JS
Pontine hypoplasia		CII, CDG, CMD, preterm disruption, JS, PCH, PTCD
Posterior fossa	Small	CII
	Enlarged	DWM
Tectal abnormality		CII, CMD, JS, RES
Vermis	Agenesis	RES, Teratogens
	Atrophy	Preterm disruption, PCH
	Dysplasia	CMD, JS
	Hypoplasia	DWM, JS, PCH, PTCD
Ventriculomegaly		Cerebellar hemorrhage, CII, CMD, CMV, DWM, RES, SLO, sinovenous thrombosis, tumor
White matter signal abnormality	Cerebellar	AGS, CS, Krabbe, MSUD, NKH, PDH, PMD
	Cerebral	AGS, CMV, CMD, CS, Krabbe, MSUD^a^, NKH^a^, PMD

^a^only myelinated white matter tracts, *AGS* Aicardi-Goutières syndrome, *BD* brainstem disconnection, *CDG* congenital disorders of glycosylation, *CII*, Chiari type II malformation, *CMD* congenital muscular dystrophy, *CMV* cytomegalovirus infection, *CNCL* congenital neuronal ceroid lipofuscinosis, *CS* Cockayne syndrome, *DWM* Dandy-Walker malformation, *HII* hypoxic-ischemic injury, *HSV* herpes simpex infection, *JS* Joubert syndrome, *MSUD* maple syrup urine disease, *NKH* nonketotic hyperglycinemia, *PCH* pontocerebellar hypoplasia, *PDH* pyruvate dehydrogenase deficiency, *PMD* Pelizaeus-Merzbacher disease, *PTCD* pontine tegmental cap dysplasia, *RES* rhombencephalosynapsis, *SLO* Smith-Lemli-Opitz syndrome, *SOD* sulfite oxidase deficiency

## Inherited cerebellar lesions

### Cerebellar malformations

#### Dandy-Walker malformation (DWM)

DWM is the most common posterior fossa malformation. It mainly occurs sporadically and has a low recurrence risk. DWM may be isolated or part of chromosomal anomalies or Mendelian disorders. Recent genetic studies suggested that DWM may be caused by signaling defects affecting the cerebellum and its overlying mesenchyme.

The dominant clinical feature of DWM is the occurrence of hydrocephalus, that typically develops during infancy [5]. Because of the progress and increasing availability of prenatal MRI, an increasing number of cases are being diagnosed in utero and during the neonatal period even in the absence of hydrocephalus. Neonates with syndromic DWM such as those with Ritscher-Schinzel syndrome or Ellis-van Creveld syndrome may present due to associated extracerebellar or systemic abnormalities.

DWM is defined by neuroimaging findings including hypoplasia, elevation, and anticlockwise rotation of the vermis and cystic dilatation of the fourth ventricle. The dilated fourth ventricle extends posteriorly filling nearly the entire posterior fossa [6]. Elevation of tentorium and torcula, enlargement of the posterior fossa, hydrocephalus, brainstem hypoplasia, and supratentorial malformations may be present.

Correction of hydrocephalus is the main treatment in DWM. Shunt placement within the lateral ventricles and/or posterior fossa cyst is currently considered the surgical treatment of choice. The long-term outcome is variable. Abnormal vermian lobulation and additional brain malformations are unfavorable predictors of cognitive outcome [7].

DWM is defined by the cerebellar involvement. While cerebellar involvement is unlikely to be responsible for the neonatal presentation, it plays an important role in clinical symptoms/findings later in life, including ataxia (present in 30–40 % patients with DWM) and cognitive dysfunction.

#### Joubert syndrome (JS)

JS has an estimated prevalence of 1:80,000, is almost always inherited with an autosomal-recessive pattern, and is caused by mutations in more than 30 genes coding for proteins of the primary cilia [8, 9].

JS may present during the neonatal period [10]. Hypotonia of variable severity is present in almost all patients. An abnormal breathing pattern including tachypnea (up to 200 breaths per minute) intermixed with apnea occurs in approximately 30 % of patients. This respiratory instability improves over time, and disappears spontaneously within the first 2 years of life. Recently, we reported that head titubation is a benign, early presentation of JS [11]. Head titubation is horizontal, presents within the first 2 months of life only when children are awake, and decreases in severity over time until it spontaneously resolves. A variety of craniofacial dysmorphic features may also be present in JS including prominent forehead, high rounded eyebrows, epicanthal folds, and open mouth. Systemic abnormalities including colobomas, retinal dystrophy, renal cysts, and polydactyly are also found during the neonatal period. The presence of enoral findings (e.g. tongue hamartoma, additional frenula, and upper lip notch) and/or mesoaxial polydactyly allows the diagnosis of oral-facial-digital syndrome type VI, a phenotype of JS. Ataxia, ocular motor apraxia, and intellectual disability are common features in JS that develop later in life.

**Fig. 1:**
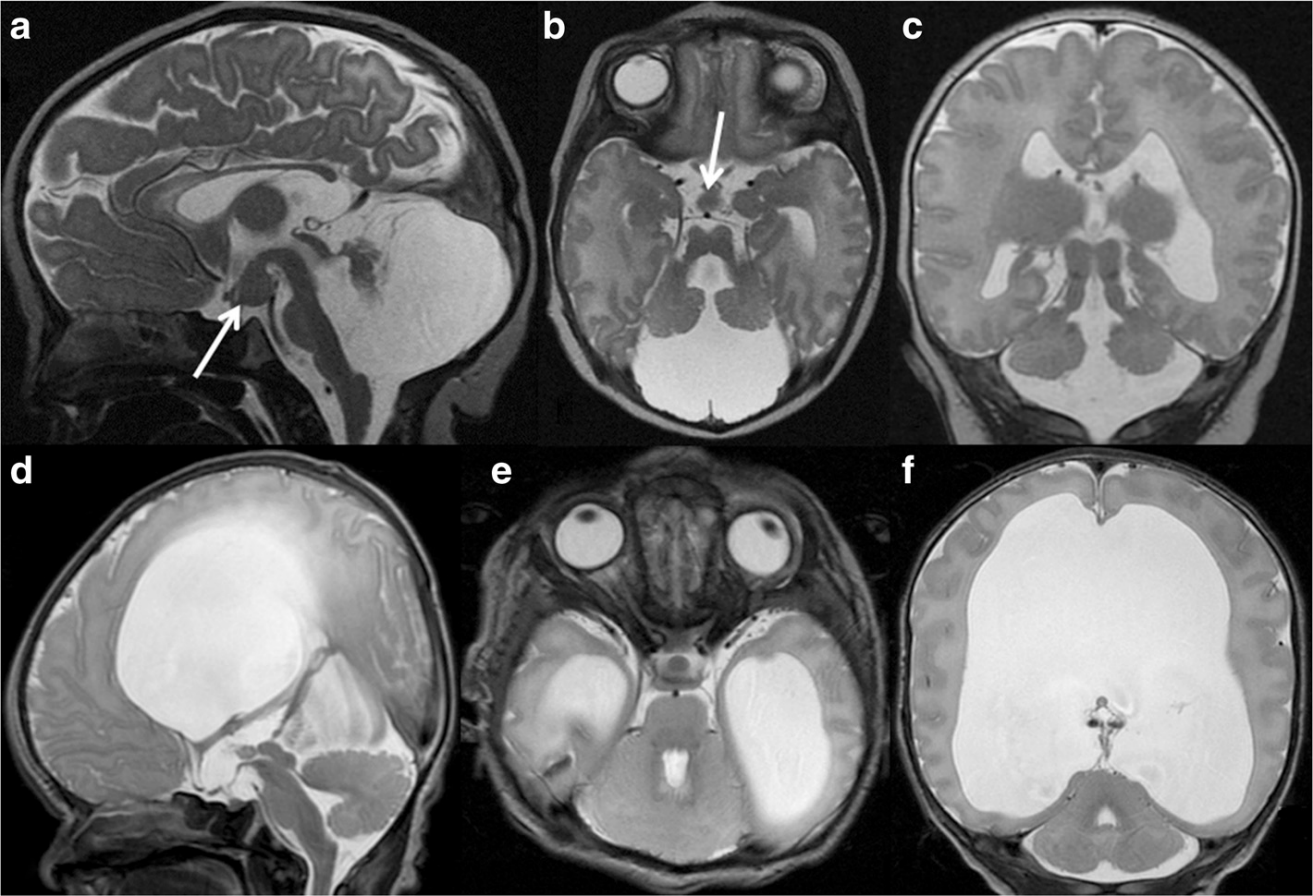
**a**, Sagittal, **b**, Axial, and **c**, Coronal T2-weighted MR images of a 2-day-old term male newborn with Oral-Facial-Digital syndrome type VI (a phenotype of Joubert syndrome) show severe hypoplasia of the cerebellar vermis and both cerebellar hemispheres, the characteristic molar tooth sign including thickened and elongated superior cerebellar peduncles and a deepened interpeduncular fossa, and a hypothalamic hamartoma (arrows)(reprinted with permission from Poretti A et al., AJNR Am J Neuroradiol, 2008;29:1090–91). **d**, Sagittal, **e**, Axial, and **f**, Coronal T2-weighted MR images of a 5-day-old term male neonate with rhombencephalosynapsis reveal continuity of the cerebellar hemispheres, dentate nuclei, and superior cerebellar peduncles without a midline intervening vermis. On the sagittal image, no primary fissure is seen. In addition, dysplasia of the tectal plate, obstruction of the Sylvian aqueduct at the level of the inferior colliculi, and marked supratentorial hydrocephalus. Note that the dentate nucleus is visible on a midsagittal image, while in normal anatomy the vermis separates the dentate nuclei in the midline

JS is defined by the presence of the molar tooth sign (MTS) on axial neuroimaging. The MTS is characterized by elongated, thickened, and horizontally orientated superior cerebellar peduncles and a deep interpeduncular fossa (Fig. 1a-c). Hypoplasia and dysplasia of the cerebellar vermis is another consistent finding. The spectrum of neuroimaging findings extends beyond the MTS and vermian hypo-dysplasia, and may include a variety of infra- and supratentorial findings [12].

The long-term outcome of JS is variable. A small number of children may die in the neonatal period or infancy for unknown causes (possibly related to breathing abnormalities). Intellectual disability is present in almost all patients, but there is a broad spectrum of severity. Liver and renal involvement may cause high morbidity and mortality and needs appropriate work-up and regular follow-up.

In JS, cerebellar involvement allows for early diagnosis and is most likely responsible for the clinical presentation during the neonatal period.

#### Rhombencephalosynapsis (RES)

RES is a sporadic malformation that is most likely caused by disruption of dorsal-ventral patterning. Children with RES typically present later in life with ataxia and intellectual disability [13]. RES, however, is commonly associated with hydrocephalus due to stenosis of the Sylvian aqueduct and affected children may present as newborns with congenital hydrocephalus. RES may occur in isolation or be part of the Gómez‐López‐Hernández syndrome, which is characterized by RES, parietal alopecia, trigeminal anesthesia, and craniofacial dysmorphic signs [14]. Alopecia is usually bilateral, bitemporal, and present from birth.

RES is defined by absence of the vermis and continuity/fusion of the cerebellar hemispheres, dentate nuclei, and superior cerebellar peduncles (Fig. 1d-f) [15]. RES may be associated with other brain anomalies such as hydrocephalus, tectal dysplasia, and callosal dysgenesis.

The long-term cognitive outcome is variable ranging from normal cognitive function to severe intellectual disability. Severe RES with associated tectal dysplasia and other cerebral anomalies is a predictor of poor cognitive outcome.

In RES, cerebellar involvement allows the diagnosis. However, it is not likely to be responsible for the neonatal presentation, but may plays a role in clinical symptoms/findings later in life (ataxia) and impaired cognition.

#### Pontocerebellar hypoplasias (PCHs)

PCHs are a group of autosomal recessive neurodegenerative disorders with a prenatal onset [16]. To date, 10 subtypes with different phenotypes and genotypes have been identified. Some forms of PCH present during the neonatal period [16]. PCH1 is characterized by loss of motor neurons in the anterior horn of the spinal cord resulting in severe hypotonia, weakness, dysphagia, and respiratory insufficiency [17]. In PCH2, neonatal presentation includes impairment of swallowing, jitteriness, and dystonia [17]. PCH4 has a severe neonatal course with hypertonia, congenital contractures, and primary hypoventilation requiring prolonged mechanical ventilation [18]. PCH6 is characterized by neonatal encephalopathy with dysphagia, seizures, generalized hypotonia, and progressive microcephaly [19]. Developmental milestones are not reached. Lactic acidemia and elevated lactate in the cerebrospinal fluid have been found in patients with PCH6 due to reduced activity of mitochondrial complexes I, III, and IV.

The cerebellar abnormality in PCH is characterized by ponto-cerebellar hypoplasia and progressive atrophy with prenatal onset [20]. In some cases of PCH (particularly type 2 due to *TSEN54* mutations) there is more severe involvement of the cerebellar hemispheres compared to the vermis. On coronal T2-weighted images, this pattern has a “dragonfly” appearance that is created by flattened cerebellar hemispheres (the “wings”) and a relatively preserved cerebellar vermis (the “body”). Absence or significant reduction of the pontine prominence is characteristic of prenatal onset in PCH. In PCH4, the cerebellar vermis is more severely affected compared to the hemispheres.

The long-term outcome is variable and depends on the underlying type of PCH. In PCH, the cerebellar and brainstem involvement may suggest the diagnosis that subsequently needs to be confirmed by genetic analysis. It is unclear how the cerebellar involvement may explain the clinical features, however brainstem involvement may be partially responsible for the movement disorders reported in PCH.

#### Congenital muscular dystrophies due to α-dystroglycanopathy

The α-dystroglycanopathies are a group of autosomal-recessive congenital muscular dystrophies resulting from mutations in more than 15 genes responsible for the O-glycosylation of α-dystroglycan [21]. The muscles, brain, and eyes are usually affected. Based on the severity of findings, various phenotypes have been described including Fukuyama disease, muscle-eye-brain disease, and Walker-Warburg syndrome, which is the most severe form [22]. Neonatal presentation is typical and includes weakness with predominant involvement of the proximal muscles, hypotonia, contractures, seizures, and macrocephaly [23]. Eye involvement is variable and may include retinal dysplasia and dystrophy, glaucoma, microophthalmia, and cataracts. Typically, creatine kinase is markedly elevated.

On neuroimaging, neonatal infratentorial involvement is characterized by cerebellar hypo-dysplasia, pontine hypoplasia, ventral pontine cleft, and ponto-mesencephalic kinking. Multiple small cortical/subcortical cerebellar cysts are common in muscle-eye-brain disease and Fukuyama disease, but develop only in the first months of life [24]. Supratentorial involvement is variable and ranges from mild ventriculomegaly, diffuse periventricular white matter changes, and focal areas of polymicrogyria to severe hydrocephalus, generalized white matter signal changes, and diffuse cortical abnormalities, including cobblestone lissencephaly [22].

The long-term outcome is variable and depends on the underlying phenotype (e.g. Fukuyama disease has the most favorable outcome) and genotype.

In α-dystroglycanopathies, the cerebellar and brainstem involvement associated with the cerebral anomalies and clinical findings may suggest the diagnosis that then needs to be confirmed by genetic analysis or muscle biopsy. The clinical presentation is most likely explained by the cerebral, muscle, and eye involvement, while the role of the cerebellar involvement in the clinical presentation remains unclear.

#### Pontine tegmental cap dysplasia (PTCD)

PTCD is a rare sporadic brainstem malformation with unknown genotype and no familial recurrence and most likely results from abnormal axonal guidance and/or neuronal migration.

Children with PTCD may present in the neonatal period with hearing loss, facial paralysis, trigeminal anesthesia, dysphagia, and reduced opening of the mouth [25, 26]. Systemic involvement with vertebral segmentation anomalies, rib malformations, and congenital heart defects has been observed.

The neuroimaging findings are pathognomonic and include a flattened ventral pons, vaulted pontine tegmentum (the “cap”), partial absence of the middle cerebellar peduncles, absence of the inferior cerebellar peduncles, vermian hypoplasia, a molar tooth-like aspect of the ponto-mesencephalic junction, and absent inferior olivary prominence [25]. The degree of brainstem dysplasia seems to correlate with the developmental disability.

The intellectual prognosis appears to be highly variable, ranging between mild cognitive delay to severe disability.

In PTCD, the brainstem involvement is diagnostic and responsible for the clinical presentation.

#### Brainstem disconnection (BD)

BD is an extremely rare abnormality of unknown etiology. All children are symptomatic at birth. The clinical manifestations are characterized by brainstem dysfunction including absent or weak sucking and swallowing, absent or markedly insufficient breathing in the majority of patients, increased or decreased muscle tone, and reduced or poor visual fixation [27]. Seizures and unstable body temperature may occur.

BD is defined by the characteristic neuroimaging pattern including nearly complete absence of a brainstem segment with the intact rostral and caudal portions connected only by a thin cord of tissue on neuroimaging [27]. BD is usually associated with cerebellar hypoplasia. Supratentorial abnormalities are unusual. In the majority of the patients, the basilar artery is missing.

The majority of children die within the first 2 months of life. Nasogastric tube or gastrostomy, intubation and mechanical ventilation, and temperature management systems are needed to treat the swallowing disorders, respiratory insufficiency, and episodic hyperthermia these infants have.

In BD, the brainstem involvement is diagnostic and responsible for the clinical presentation.

#### Chiari type II malformation (CII)

CII is not a cerebellar malformation, but a spinal dysraphism with secondary morphological anomalies of the posterior fossa contents. Nevertheless, we decided to include CII in this group of diseases. CII occurs in about 1 of 1000 live births and is universally associated with non skin-covered myelomeningocele/spinal dysraphia [28].

Affected neonates are symptomatic during the neonatal period due to spinal involvement and hydrocephalus. Clinical features of CII related to cerebellar and brainstem anomalies, are present in approximately 30 % of neonates, and include dysphagia, vocal cord paralysis with laryngeal stridor, and central or obstructive ventilation abnormalities with apneic episodes. The presence of stridor, apnea, cyanotic spells, and dysphagia is associated with increased mortality [29].

CII is defined by a small posterior fossa with inferior displacement of the lower cerebellum, medulla, and fourth ventricle through the foramen magnum [28]. Hydrocephalus is present in up to 90 % of affected neonates and is caused by the hindbrain malformation that blocks/impairs the cerebrospinal fluid hydro-dynamics of the posterior fossa or by aqueductal stenosis. Additional morphological abnormalities include beaking of the tectal plate, pontine hypoplasia, callosal dysgenesis, large massa intermedia, and cortical malformations [30].

Intrauterine surgical repair of the myelomeningocele between 19 and 25 weeks of gestation has been shown to reduce the rate of shunt placement, improve motor function at 30 months, and decrease severity of hindbrain herniation [31, 32].

In CII, the cerebellar and brainstem findings are diagnostic and partially responsible for the clinical presentation.

### Neurometabolic disorders with neonatal cerebellar involvement

#### Congenital disorders of glycosylation (CDG)

CDG include nearly 50 inborn errors of glycan metabolism with an estimated prevalence of 1:20,000 [33]. This rapidly growing group of disorders encompasses defects in N-glycosylation, O-glycosylation, and lipid-linked glycosylation. The most common congenital disorder of glycosylation is phosphomannomutase II deficiency (PMM2-CDG) [33].

In these diseases, there is significant phenotypic diversity. Classically babies present with multi-system involvement including abnormal fat distribution, inverted nipples, hypotonia, hyporeflexia, and strabismus [33]. Ataxia may develop in some patients. In PMM2-CDG, brain MRI may be normal, however, MRI often reveals significant pontocerebellar hypoplasia and superimposed cerebellar atrophy and T2-hyperintense signal of the cerebellar cortex [34]. Cerebellar atrophy and T2-hyperintense signal of the cerebellar cortex are typically not present in the neonatal period, but may develop later.

In CDG, pontocerebellar hypoplasia may suggest the diagnosis, which must then be confirmed by transferrin electrophoresis and genetic analysis. Pontocerebellar hypoplasia may explain the occurrence of ataxia in CDG. Treatment for most CDG remains symptomatic and palliative. The prognosis of CDG is variable and depends on the underlying phenotype and genotype.

#### Smith-Lemli-Opitz syndrome (SLO)

SLO has an estimated prevalence of around 1:20,000–40,000 births and results from abnormal cholesterol biosynthesis [35]. Patients typically have dysmorphic features, which can include a broad nasal bridge, anteverted nares, and long philtrum. Skeletal abnormalities are common and most patients have syndactyly of the 2nd and 3rd toes. A variety of systems can be involved and neonates can have cardiovascular, gastrointestinal, renal, and genitourinary abnormalities. Neurologic manifestations include congenital microcephaly, ptosis, and hypotonia [35]. Structural brain abnormalities are seen in about one-third of patients and include ventriculomegaly, hypoplastic frontal lobes, absent or hypoplastic corpus callosum, and, in some patients, cerebellar hypoplasia [36].

The diagnosis of SLO is based on the detection of elevated 7-dehydrocholesterol in blood or tissues and can be confirmed by mutation analysis. The blood cholesterol level is not a reliable screening test. Management is symptomatic. Long-term outcome is variable and depends on the severity of the disease and associated malformations. Cardiovascular and brain malformations may be lethal.

Cerebellar involvement with global cerebellar hypoplasia is inconsistently present and non-specific.

#### Nonketotic hyperglycinemia (NKH)

NKH has an estimated prevalence of around 1:55,000–65,000 births and results from a deficiency in glycine cleavage with subsequent toxic systemic accumulation of glycine. Central nervous system toxicity results and infants typically present in the first 2 days of life with depressed alertness, hypotonia, myoclonic jerks, and refractory seizures. Many progress quickly to apnea, coma, and death [37]. EEG commonly reveals a burst suppression pattern. Early neuroimaging findings include restricted diffusion of myelinated white matter tracks (including cerebellar and brainstem white matter tracts), agenesis of the corpus callosum, and a glycine peak on ^1^H-MR-spectroscopy [36]. Hypoplasia of the cerebellar vermis is seen in a minority of patients [37].

The diagnosis is established by an increase in CSF glycine concentration and an increased CSF-to-plasma glycine ratio, and can be confirmed by genetic analysis.

Sodium benzoate may be used to reduce plasma glycine levels. Otherwise management is primarily symptomatic. Long-term outcome is usually poor and early death may occur due to apnea.

Cerebellar involvement with vermis hypoplasia is inconsistently present and non-specific. ^1^H-MR-spectroscopy showing a glycine peak may facilitate the diagnosis of NKH.

#### Maple syrup urine disease (MSUD)

MSUD is an autosomal recessive condition that results from a deficiency in branched-chain α-ketoacid dehydrogenase enzyme and causes an accumulation of leucine, isoleucine, and valine. Leucine and its byproducts exert the primary neurotoxic effects resulting in intramyelinic edema. MSUD has an estimated prevalence of 1:150,000 live births.

**Fig. 2:**
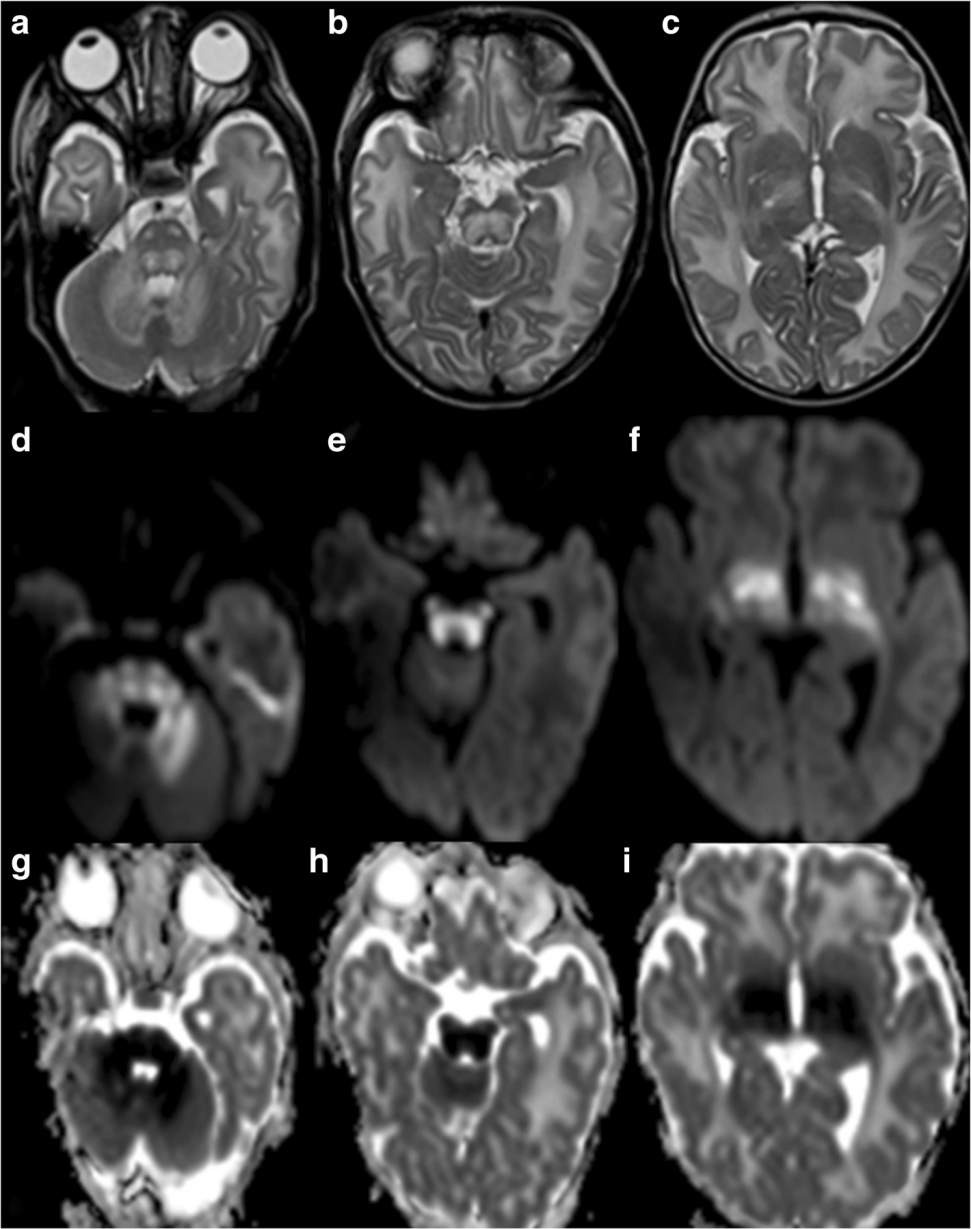
**a**-**c**, Axial T2-weighted MR images of a 20-day-old male term neonate with maple syrup urine disease show swelling and hyperintense signal of the cerebellar white matter, dorsal pons, corticospinal tracts along its course in the basis pontis, midbrain, and posterior limbs of the internal capsule, and thalami. **d**-**f**, Trace of diffusion images and **g-i**, Apparent diffusion coefficient (ADC) maps of the same newborn reveal bright DWI-signal and matching low ADC values, respectively, in the cerebellar white matter, dorsal pons, corticospinal tracts in the basis pontis, midbrain, and posterior limb of the internal capsule, and thalami representing restricted diffusion/cytotoxic edema compatible with extensive ongoing injury to the myelinated parts of the brain

Neonates typically present in the first week of life with poor feeding, recurrent emesis, lethargy, fluctuating ophthalmoplegia, and seizures [38]. Many newborns develop dystonia and stereotyped, “bicycling” or “fencing” movements of the extremities [38]. During the acute phase of deterioration, neuroimaging shows T2-hyperintense signal and decreased diffusion of the cerebellar and other white matter regions that are already myelinated (Fig. 2) [36, 39].

The diagnosis is confirmed by detecting increased values of branched-chain amino acids (leucine, isoleucine, and valine) and branched-chain keto-acids in blood and urine. Early diagnosis is paramount for a favorable long-term outcome; children may be able to achieve a normal cognitive outcome if appropriate intervention (diet) occurs early in the neonatal course [40].

In MSUD, cerebellar involvement on neuroimaging is distinctive and may be helpful in establishing an early diagnosis.

#### Pyruvate dehydrogenase deficiency (PDH)

PDH results from mutations in the pyruvate dehydrogenase complex, which is responsible for the conversion of pyruvate to acetyl-CoA. In the neonatal form, patients typically present with hypotonia, encephalopathy, microcephaly, and tachypnea [41]. Approximately one-third develop seizures. MRI findings include dysgenesis of the corpus callosum, mega cisterna magna, heterotopias, pachygyria and cortical atrophy. T2-hyperintense signal and decreased diffusion can occur in the cerebellar white matter, posterior limb of the internal capsule, and occipital lobes. ^1^H-MR-spectroscopy may reveal a doublet peak at 1.3 ppm compatible with lactate [36].

Lactate is typically elevated in the blood and CSF. The diagnosis is suspected by abnormal enzyme function and can be confirmed by genetic analysis. Treatment typically includes cofactor supplementation (e.g. thiamine and carnitine) and ketogenic diet. Long-term outcome is generally poor.

Cerebellar involvement with T2-hyperintense signal and restricted diffusion in the cerebellar white matter is non-specific.

#### Sulfite oxidase deficiency (SOD)

Isolated SOD and SOD due to molybdenum cofactor deficiency result in the accumulation of sulfites, which are toxic to the developing brain [42]. SOD may present with poor feeding, emesis and seizures [43]. The onset of seizures is typically accompanied by rapid neurologic deterioration [44]. Dysmorphic features are present in up to 75 % of patients, including coarse facies, small nose, elongated face, and long philtrum [42, 43]. Ectopia lentis is present later in the disease course [43]. Initial neuroimaging with MRI reveals generalized white matter edema, mimicking hypoxic ischemic injury [36].

The diagnosis is based on high sulfite, thiosulfate, S-sulfocysteine, and taurine concentrations in urine and should be confirmed by genetic analysis. The long-term outcome is usually poor including intractable seizures and death in early infancy. Substitution therapy with purified cyclic pyranopterin monophosphate is a new, promising causative therapy for molybdenum cofactor deficiency [45].

Cerebellar involvement with global cerebellar hypoplasia is inconsistently present and non-specific [36].

### Neurodegenerative disorders with neonatal cerebellar involvement

#### Krabbe disease (KD)

KD has an estimated prevalence of 1:100,000 and is caused by deficiency of the lysosomal enzyme galactocerebrosidase and is characterized by failure of myelination in the central and peripheral nervous systems [46]. Neonatal presentation of KD is characterized by spasticity, marked irritability, and lack of motor development [47]. Neuroimaging shows signal abnormalities of the lateral thalami, corona radiata, cerebellar white matter, and dentate nuclei.

In KD, cerebellar involvement is characteristic and may suggest the diagnosis, which needs to be confirmed by low galactocerebrosidase activity in peripheral leukocytes and genetic analysis. Treatment is symptomatic. Long-term prognosis is poor and neurodegeneration and early death occurs within the first 2–3 years of life.

#### Cockayne syndrome (CS)

CS is a nucleotide excision repair disease with an estimated prevalence of 1:200,000. CS may present during the neonatal period (CS type II) with prenatal and postnatal growth failure, congenital cataracts, loss of adipose tissue, joint contractures, and distinctive face with small, deep-set eyes and prominent nasal bridge [48]. Myelination is disturbed within the central and peripheral nervous system. Additional brain involvement includes basal ganglia calcifications and severe cerebral and cerebellar atrophy.

In CS type II, cerebellar atrophy is a non-specific finding. The entire clinical and imaging findings may suggest the diagnosis that needs confirmation by genetic analysis.

Management is supportive and the progressive course leads typically to death before the end of the first decade.

#### Pelizaeus-Merzbacher disease (PMD)

**Fig. 3:**
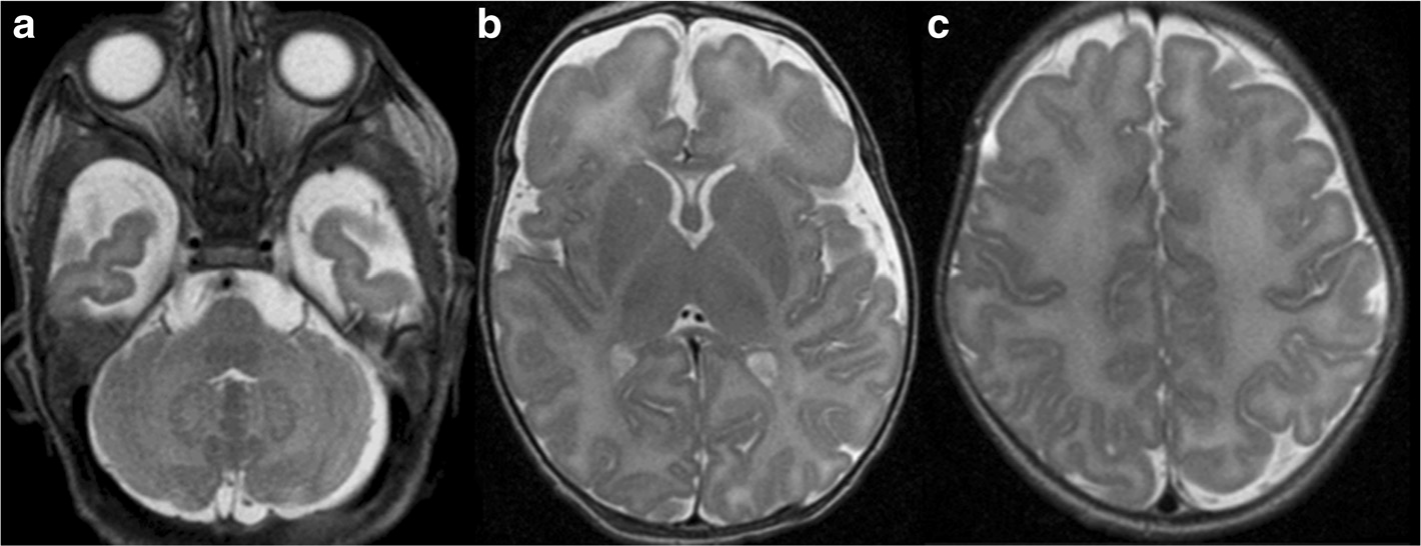
**a**-**c**, Axial T2-weighted MR images of a 5-day-old male term neonate with genetically confirmed Pelizaeus-Merzbacher disease presenting with marked muscular hypotonia, rotatory nystagmus, and inspiratory stridor show a homogeneous increased hyperintense signal of the supra- and infratentorial white matter including the corticospinal tracts within the posterior limb of the internal capsule bilaterally compatible with hypomyelination

PMD is a hypomyelinating disorder that is caused by *PLP1* mutations and has an estimated prevalence of 1:400,000 [49]. PMD may present during the neonatal period (connatal PMD) with hypotonia, inspiratory stridor, horizontal or rotatory nystagmus, and seizures. Neuroimaging shows near complete absence of central nervous system myelin including brainstem and cerebellar white matter tracts (Fig. 3).

In PMD, complete absence of myelin within the brainstem and cerebellar white matter associated with the clinical findings may suggest the diagnosis, which then needs to be confirmed by genetic analysis. Management is symptomatic. PMD has a progressive course leading to death by the second decade.

#### Aicardi-Goutières syndrome (AGS)

AGS is a genetically heterogeneous inflammatory disease and is an important diagnostic consideration in neonates with imaging findings suggestive of perinatal infection. AGS is consistently associated with an interferon signature, which is sustained over time and can be used for diagnostic purposes. Newborns with AGS classically have intracranial calcifications, white matter abnormalities, and cerebral atrophy. Calcifications can be seen in the dentate nuclei in approximately 30 % of affected children [50]. In addition, cerebellar atrophy has been reported inconsistently as a late feature.

In AGS, dentate nuclei calcifications as well as other neuroimaging findings and clinical features may suggest the diagnosis that needs confirmation by genetic analysis. Treatment is symptomatic and the majority of patients presenting early in life die within the first 10 years of life.

#### Congenital neuronal ceroid lipofuscinosis (CNCL)

CNCL is a rare neurodegenerative disease caused by mutations in the *Cathepsin D* gene [51]. The neonatal presentation is severe and includes therapy-resistant seizures, respiratory insufficiency, and marked microcephaly. Neuroimaging findings are characterized by severe cerebral and cerebellar atrophy. Absence of electro-retinographic responses and iso-electric electroencephalography are characteristic.

In CNCL, severe neonatal cerebellar atrophy associated with the above imaging and clinical findings may suggest the diagnosis that then needs to be confirmed by genetic analysis. The neonatal clinical features are most likely caused by severe cerebral atrophy. Failure of neurological development and development of a vegetative state are usually followed by death within the first weeks of life.

### Spinocerebellar ataxias (SCAs) with neonatal cerebellar involvement

SCAs are a clinically heterogeneous group of disorders with a global prevalence of 1–4/100.000 [52]. More than 40 different types of SCAs have been described and they are inherited with an autosomal-dominant pattern [53]. Various SCAs types are so called trinucleotide repeat disorders that are mostly caused by expansions of CAG repeats. SCAs typically present in adults and are rare causes of childhood ataxia. In rare cases, SCA2 and SCA7 may present in the neonatal period.

SCA2 is caused by an expansion of a CAG trinucleotide repeat near the 5′ coding region of the *ataxin 2* gene on chromosome 12q24. There is an inverse correlation between the repeat size and the age of onset: full penetrance alleles are usually 37–39 in adult cases, while very large expansions of over 200 have been reported in infants [54]. The neonatal presentation of SCA2 is characterized by hypotonia, absent reflexes, dysphagia, poor head control, and lack of visual fixation [55, 56]. In the first year of life, infants with SCA2 usually develop developmental delay or regression, progressive encephalopathy, optic atrophy, retinitis pigmentosa, and seizures [54–56]. Neuroimaging findings typically include cerebellar atrophy, mild reduction in size of the pons, and progressive volume loss of the cerebral white matter with secondary ventriculomegaly and thinning of the corpus callosum, but brain MRI may reveal normal findings [54].

SCA7 is caused by expansion of a CAG trinucleotide repeat in the first coding exon of the *ataxin 7* gene located on chromosome 3p. There is an inverse correlation between the size of the repeat and age of onset. The adult and childhood phenotype is characterized by visual loss due to retinitis pigmentosa, progressive ataxia, dysmetria, intention tremor, dysarthria, dysphagia, and brisk reflexes. The neonatal phenotype is quite different and includes failure to thrive, weight loss, weakness, hypotonia, and acquired microcephaly [57, 58]. Patients may have a patent ductus arteriosus and evidence of multiple organ failure. In the first year of age, infant with SCA7 may develop neurological deterioration and retinal dystrophy. Neuroimaging findings are characterized by cerebellar and cerebral atrophy with secondary ventriculomegaly.

### Acquired cerebellar lesions

#### Disruption of cerebellar development in preterms

Cerebellar injuries in premature infants may be grouped into two categories: 1) primarily destructive injuries including hemorrhage and ischemia (as discussed later) and 2) injuries primarily impairing cerebellar development [59]. The latter may represent the most common type of cerebellar abnormality of the premature infant. Two pathomechanisms are possible: 1) direct effects on the rapidly growing/developing cerebellum and 2) remote effects through altered trophic trans-synaptic interactions.

Direct effects on the growing cerebellum may include hypoxic-ischemic injury or toxic effect of glucocorticoids (as discussed later), blood products, and undernutrition. In preterm neonates with intraventricular hemorrhages, hemosiderin deposition on the cerebellar surface and a progressive reduction in size of cerebellum (particularly the cerebellar hemispheres) and pons may occur [60]. Hemosiderin is likely toxic for the granule precursor cells of the external granular layer and their impaired survival and/or proliferation may cause cerebellar underdevelopment [59].

Remote trans-synaptic effects that primarily affect neuronal connections between cerebrum and cerebellum may lead to cerebellar underdevelopment in preterm neonates [61]. Disruption of cerebellar development in preterm neonates is frequently associated with contralateral periventricular leukomalacia (PVL) and other cerebral lesions raising the possibility that cerebellar underdevelopment may be secondary to the loss of supratentorial white matter. Unilateral cerebral brain injuries are associated with significantly decreased volume of the contralateral cerebellar hemisphere. Conversely, unilateral cerebellar injuries are associated with significantly decreased volume in specific regions of the contralateral cerebral hemisphere (dorsolateral prefrontal, premotor, sensorimotor, and midtemporal gray and white matter) [62]. In addition, infants with bilateral diffuse PVL typically have symmetric bilateral decreases in cerebellar volume.

In the neonatal period, the cerebellar abnormality is most often clinically silent, making this group of disorders difficult to diagnose. Symptoms and signs related to the direct effect of intraventricular hemorrhage or a remote cerebral injury may provide clues to the provider. Examples include seizure, bulging fontanelle, alteration of consciousness, or focal neurologic deficit.

**Fig. 4:**
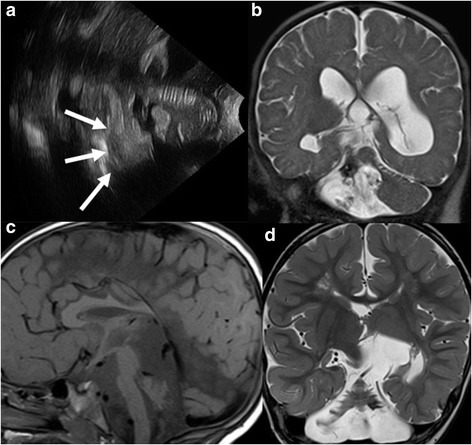
**a**, Ultrasonography image through the mastoid fontanel of a 14-day-old neonate born at 26 weeks of gestation show a cerebellar hemorrhage (arrows). **b**, Coronal T2-weighted image at 2 months of life reveals encephalomalacic changes within the right cerebellar hemisphere with T2-hypointense foci representing deposition of blood products. **c**, Sagittal T1- and **d**, Coronal T2-weighted MR images of a 3-year-old boy born at 25 weeks gestation reveal a small posterior fossa, marked reduction in the size of the cerebellar hemispheres, which have a skeletonized appearance and appear more affected compared to the small vermis. Together with the pontine hypoplasia, constellation of findings suggest disruption of the cerebellar development as a sequela of prematurity. In addition, a T2-hypointense signal is noted in the left cerebellar folia suggesting hemosiderin deposition due to remote hemorrhage. Finally, a thinned corpus callosum and encephalomalacic changes in the supratentorial brain are also seen

On conventional MRI, following morphologic patterns of disrupted cerebellar development may be seen: 1) symmetric volume reduction of the cerebellar hemispheres and a small vermis with preserved shape (Fig. 4c-d), 2) symmetric reduction in hemispheric volume with an enlarged, balloon-shaped fourth ventricle and a small, deformed vermis, and 3) normal overall cerebellar shape with extensive reduction of its dimensions [60]. Pontine hypoplasia is consistently seen. In addition, unilateral cerebellar hypoplasia may be seen secondary to contralateral PVL.

In ex-preterm neonates with cerebellar underdevelopment, impairments in motor, cognitive, and behavioral (socialization difficulties, early autistic features) functions are highly prevalent [63, 64]. Cerebellar volume has been shown to predict motor and cognitive performance in ex-preterm adolescents [64].

### Vascular disorders of the neonatal cerebellum

#### Cerebellar hemorrhages

Cerebellar hemorrhages are common neonatal acquired cerebellar injuries, particularly in preterm neonates. The prevalence depends on the degree of prematurity and birth weight. In a large series of cases, the prevalence of cerebellar hemorrhages was 2.7 % in preterm neonates with a birth weight above 750 g and 8.7 % in those below 750 g [65].

Cerebellar hemorrhages typically originate from the cerebellar germinal matrix within the external granular layer that is thickest at 24 weeks of gestation and begins to involute at 30 weeks [66]. Therefore, 21 through 27 weeks gestation is the most vulnerable time period for cerebellar hemorrhages. Less commonly, hemorrhages may originate in the residual germinal matrix of the ventricular zone in the roof of the fourth ventricle and primarily involve the vermis.

The pathogenesis of cerebellar hemorrhages is multifactorial. Different risk factors have been reported for the preterm and term neonates. In preterm neonates, maternal, intrapartum, and early postnatal characteristics are known risk factors. These include assisted conception, abnormal fetal heart rate, C-section delivery, lower Apgar scores, need for pressor support and high frequency ventilation, presence of a persistent ductus arteriosus, low pH, low plasma bicarbonate, low platelets, and low hematocrit values [65, 67]. In term neonates cerebellar hemorrhages are primarily associated with traumatic delivery [68].

Large cerebellar hemorrhages (>1 cm) may present with apnea, bradycardia, full fontanelle, or opisthotonus [68]. The majority of cerebellar hemorrhages are clinically silent in the neonatal time period and require a high index of suspicion to make the diagnosis [65–67].

The traditional ultrasonographic approach through the anterior fontanelle has a low sensitivity for detecting cerebellar hemorrhages because of the echogenic tentorium and the limited penetration depth of the ultrasound waves [65]. The advent of mastoid fontanel and suboccipital views significantly increased the sensitivity of ultrasonography for detection of cerebellar hemorrhage [65–67]. However, a high number of cerebellar hemorrhages remain undetected by ultrasonography compared to MRI [67, 69]. Brain MRI including susceptibility-weighted imaging (SWI) is the diagnostic tool of choice to detect cerebellar hemorrhages (Fig. 4a-b).

Cerebellar hemorrhages occur most commonly in only one cerebellar hemisphere (71 %). The vermis is involved in 20 % of patients, and associated supratentorial lesions are found in up to 77 % of cases [65, 67]. Focal atrophy of the affected hemisphere with a secondary reduction in size of the pons may be seen approximately 2 months later in as many as 37 % of patients [65]. Interestingly, significant volume reduction of the contralateral cerebral hemisphere may be present and most likely results from impaired remote trans-synaptic neurodegeneration [65].

In preterm neonates, cerebellar hemorrhages result in high mortality (14 % compared to 1 % of control preterms) and long-term neurological morbidity (66 % compared to 5 % of control preterms) [65, 70]. Neurological sequelae include motor dysfunction in 48 % (hypotonia in 100 %, abnormal gait in 37 %, and ocular motor abnormalities in 23 %), expressive and receptive language dysfunction in 37 % to 42 %, and neurocognitive dysfunction in 40 % of children [70]. Additionally, autistic features may become apparent in up to 42 %, and more than 30 % have internalizing behavioral problems. Lesions within the cerebellar hemispheres have been associated with impaired cognitive function, while injury of the cerebellar vermis may cause motor, language, and behavioral/social problems. In term neonates, the neurological sequelae are similar, but less prevalent than in preterms [68].

#### Sinovenous thrombosis with cerebellar involvement

Cerebral sinovenous thrombosis (CSVT) affects 2.6–12/100,000 neonates and is defined by thrombosis within the superficial or deep venous system [71]. In neonates, 39 % of CSVT involve the transverse sinuses, and 30 % involve the straight sinus [72]. In these cases, thrombosis may lead to venous congestion and secondary venous infarction of the hemispheric white matter with or without hemorrhagic conversion of the ischemic cerebellum.

Generally, many risk factors for neonatal CSVT have been reported [72–74]. However, no specific risk factors have been reported for CSVT affecting the posterior fossa. Clinically, CSVT tends to present with seizure or decreased alertness rather than a focal neurologic sign. The presentation may be subtle and some neonates may have isolated irritability [72]. No specific symptoms have been reported for CSVT affecting the cerebellum. A high index of suspicion is required to make the diagnosis.

Brain MRI with MR venography is the neuroimaging modality of choice [73]. In addition, diffusion weighted imaging (DWI) and SWI may be helpful to identify ischemic and/or hemorrhagic complications. DWI may also be helpful to directly visualize the intravascular thrombus. A hypercoagulability evaluation should be performed to identify risk factors for future thromboses. Treatment is controversial and varies by institution; there are studies that indicate that anticoagulation may be safe, and may decrease the risk of clot propagation [74].

Overall the outcome of CSVT is variable: 45–64 % of the affected neonates have a normal outcome, 3–19 % die, 16–18 % have seizures (higher risk with thalamic injury), and 33–65 % have neurologic deficits including both motor and cognitive deficits [72, 73]. No specific outcomes have been reported for neonates with CSVT affecting the cerebellum.

#### Cerebellar ischemic stroke

In neonates, cerebellar ischemic stroke is very rare and less common than cerebellar hemorrhages [68]. Some hypothesize that neonatal ischemic stroke is responsible for damage involving mostly the inferior parts of the cerebellum with secondary pontine hypoplasia in very preterm neonates [75, 76]. The consistent association of cerebellar lesions with cerebral white matter injury may suggest a similar pathogenesis of both lesions (e.g. global hypoxia/ischemia or infection/inflammation rather than vascular occlusive). Alternatively, injuries primarily impairing cerebellar development (discussed above) may be to blame.

In the neonatal period, ischemic cerebellar stroke is most often clinically silent, but apnea and seizures may occur. Brain MRI with DWI is the diagnostic neuroimaging modality of choice.

### Hypoxic-ischemic injury (HII)

HII affects 1–3 newborns per 1000 full-term births in the United States [77]. Depending on the severity and timing of injury, four neuroimaging patterns involving cerebral cortex/white matter, usually in a parasagittal distribution, deep nuclear structures, especially putamen and thalamus, and brainstem tegmentum have been reported [77]. Though literature about cerebellar involvement in HII is scant, animal models have shown that Purkinje cells are highly vulnerable to HII due to their inability to generate energy during hypoxia [78]. The relative immaturity of the neonatal Purkinje cells in the cerebellar cortex is thought to have a protective effect on the cerebellar gray matter in HII [79].

In children with HII and cerebellar involvement, clinical cerebellar signs have not been reported. This is not surprising, given neonates with cerebellar involvement typically have extensive supratentorial injuries [80]. In these patients, the clinical presentation is dominated by features such as seizures and encephalopathy that result from supratentorial injury.

In neonatal HII, acute injury of the cerebellum is rarely depicted on conventional MRI sequences (3–4 % of the patients), but common on neuropathology exam of the brains of asphyxiated newborns who died (up to 70–80 %) [80]. This difference suggests that cerebellar involvement is most likely present in neonates with severe HII. Alternatively, cerebellar injury may be subtle and difficult to identify on conventional MRI sequences. The application of diffusion tensor imaging (DTI) showed reduced factional anisotropy and increased apparent diffusion coefficient values in the cerebellar peduncles of asphyxiated neonates [80]. Interestingly, all neonates who had changes in DTI scalars in the cerebellar tracts also had injury to the supratentorial brain. This supports the idea that cerebellar injury in HII occurs predominantly in severely asphyxiated neonates with cerebral injury. The association between supratentorial (particularly basal ganglia and thalami) and cerebellar abnormalities in HII has also been shown on follow-up MRI studies [81, 82]. Involvement of both 1) basal ganglia and thalami and 2) cerebellum raises the possibility that cerebellar injury in HII may be secondary to transsynaptic degeneration via the thalamo-cerebellar pathway [81]. On follow-up studies in HII, selective involvement of the anterior part of the cerebellar vermis is seen as a hyperintense signal on T2-weighted images and/or selective atrophy [81, 82]. In addition, minimal subsequent growth of the cerebellar vermis has been shown in infants with HII and severe basal ganglia and thalamic lesions [83]. Vermian involvement may serve as an independent predictor of severe HII associated with significant neurologic disability [83].

### Infectious etiologies with neonatal cerebellar involvement

**Fig. 5:**
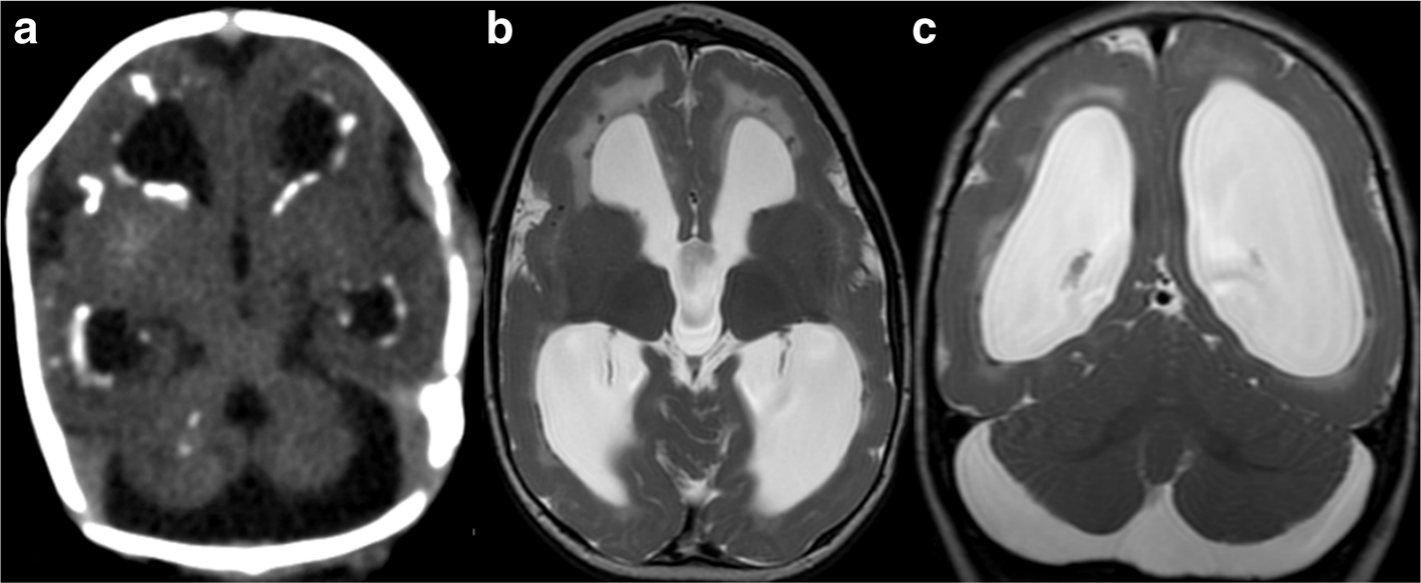
**a**, Axial computed tomography (CT) image of a 2-day-old male neonate with confirmed congenital cytomegalovirus infection shows ventriculomegaly, cerebellar hypoplasia, and periventricular hyperdense calcifications. **b**, Axial and **c**, Coronal T2-weighted MR images of the same child at the age of 14 months reveal cerebellar hypoplasia, ventriculomegaly, hyperintense signal of the periventricular white matter, hypointense periventricular calcifications, and diffuse polymicrogyria and pachygyria (reprinted with permission form Poretti A et al., Eur J Paediatr Neurol, 2009;13:397–407)

Isolated cerebellar involvement in fetal and neonatal infection is rare. While cytomegalovirus infection classically results in periventricular calcifications, a number of additional brain abnormalities can be seen, including cerebellar hypoplasia (Fig. 5) [84]. The presence of cerebellar hypoplasia suggests an early (first trimester) transmission of cytomegalovirus, which interferes with early brain development. Recently, Oosterom et al. showed that severe structural abnormalities including cerebellar hypoplasia are associated with poor neurodevelopmental outcome and/or sensorineural hearing loss [85]. It is unclear, however, how much cerebellar hypoplasia may contribute to poor neurodevelopmental outcome compared to other structural abnormalities such as polymicrogyria.

Imaging findings in neonatal herpes simplex virus (HSV) encephalitis are variable. Imaging often reveals multifocal areas of diffusion restriction (cytotoxic edema); however, cases of isolated brainstem and cerebellum involvement have been described in HSV-2 [86].

### Extrinsic neurotoxic agents with neonatal cerebellar involvement

The neonatal cerebellum has been shown to have the highest number of glucocorticoid receptors in the brain [87]. Antenatally, glucocorticoids are often given to hasten lung maturation. Postnatally, glucocorticoids may be administered for prolonged hypotension and bronchopulmonary dysplasia. Multiple animal models have demonstrated that glucocorticoids delay the growth of the cerebellum in distinct species [88]. Preterm studies have demonstrated similar findings in humans, with one group reporting a 20 % reduction in cerebellar volume in patients receiving dexamethasone, as compared to control subjects [89]. In one series of 172 serially imaged premature infants, postnatal betamethasone and hydrocortisone were associated with decreased cerebellar volumes of 10 % and 8 %, respectively. In the same cohort, antenatal betamethasone treatment was not associated with a change in cerebellar volume [90]. A smaller cerebellar volume does not seem to affect the clinical presentation of the affected premature infants. Follow-up studies at school age are needed to detect the long-term motor and cognitive consequences of a smaller cerebellar volume due to postnatal betamethasone and hydrocortisone administration.

### Congenital cerebellar tumors

Congenital brain tumors are rare and represent only 0.5–1.5 % of all pediatric brain tumors [91]. Teratomas are the most frequent congenital brain tumors, while non-teratomatous neoplasms including choroid plexus papilloma, medulloblastoma, and craniopharyngioma are less common. Up to 70 % of the tumors originate within the supratentorial brain, while the remaining 30 % are located in the posterior fossa. Macrocrania is the most common presentation; the open sutures and cartilaginous character of the fetal/neonatal skull allow significant expansion in utero. Hydrocephalus, intratumoral hemorrhage, heart failure and hydrops resulting from high-cardiac output, and polyhydramnios are less common presentations. The diagnosis of a cerebellar tumor is made by a combination of conventional and advanced neuroimaging, and histological exam is needed to characterize the exact tumor type and histology. Prognosis in neonates is generally poor, but depends on the histological type, extension of the tumor, and involvement of adjacent structures.

## Conclusion

As illustrated in this review, the cerebellum is involved in a broad range of pathologies seen in the newborn period. In many cases, characterization of cerebellar involvement can facilitate diagnosis, guide management, and improve the ability to provide an accurate prognosis for the infant. While the cerebellum is a relatively small structure anatomically, it plays a critical role in overall neurodevelopment.
